# Artificial intelligence in the diagnosis and management planning of bicuspid aortic valvular disease: a case series

**DOI:** 10.1093/ehjcr/ytaf449

**Published:** 2025-09-18

**Authors:** Tommaso Viva, Alessandro Masini, Michele Gallazzi, Vito Domenico Bruno, Antonio Miceli, Mattia Glauber, Daniele Andreini, Edoardo Conte

**Affiliations:** Department of Clinical Cardiology and Cardiovascular Imaging, IRCCS Galeazzi-Sant’Ambrogio Hospital, Via Cristina Belgioioso, 173, Milan 20157, Italy; Department of Clinical Cardiology and Cardiovascular Imaging, IRCCS Galeazzi-Sant’Ambrogio Hospital, Via Cristina Belgioioso, 173, Milan 20157, Italy; Department of Biomedical and Clinical Sciences, University of Milan, Via Festa del Perdono, 3, Milan 20100, Italy; Department of Clinical Cardiology and Cardiovascular Imaging, IRCCS Galeazzi-Sant’Ambrogio Hospital, Via Cristina Belgioioso, 173, Milan 20157, Italy; Department of Biomedical and Clinical Sciences, University of Milan, Via Festa del Perdono, 3, Milan 20100, Italy; Minimally Invasive Cardiac Surgery Unit, IRCCS Ospedale Galeazzi-Sant’Ambrogio, Via Cristina Belgioioso, 173, Milan 20157, Italy; Minimally Invasive Cardiac Surgery Unit, IRCCS Ospedale Galeazzi-Sant’Ambrogio, Via Cristina Belgioioso, 173, Milan 20157, Italy; Minimally Invasive Cardiac Surgery Unit, IRCCS Ospedale Galeazzi-Sant’Ambrogio, Via Cristina Belgioioso, 173, Milan 20157, Italy; Department of Clinical Cardiology and Cardiovascular Imaging, IRCCS Galeazzi-Sant’Ambrogio Hospital, Via Cristina Belgioioso, 173, Milan 20157, Italy; Department of Biomedical and Clinical Sciences, University of Milan, Via Festa del Perdono, 3, Milan 20100, Italy; Department of Clinical Cardiology and Cardiovascular Imaging, IRCCS Galeazzi-Sant’Ambrogio Hospital, Via Cristina Belgioioso, 173, Milan 20157, Italy

**Keywords:** Case series, Bicuspid aortic valve, Echocardiography, Artificial intelligence

## Abstract

**Background:**

Bicuspid aortic valve (BAV) is the most common congenital heart anomaly, often leading to significant aortic stenosis (AS) or aortic regurgitation (AR), which may require surgical intervention. Echocardiography is typically used for the diagnosis of BAV, and the integration of artificial intelligence (AI) can enhance diagnostic accuracy and guide surgical decisions.

**Case summary:**

We present two patients with BAV: a 17-year-old male football player with isolated AR due to prolapse undergoing aortic valve repair and a 68-year-old male with combined AS and AR, candidate for aortic valve replacement. Artificial intelligence-based tools assisted in characterizing the valvular disease and assessing its haemodynamic impact by estimating and averaging transvalvular gradients and velocity-time integrals, reconstructing three-dimensional valve anatomy, and automatically calculating left ventricular volumes, ejection fraction, and global longitudinal strain. This comprehensive assessment improved prognostic evaluation and helped tailor the treatment plan.

**Conclusion:**

Artificial intelligence in echocardiography holds great potential for diagnosis and planning the treatment of BAV disease. By enhancing image analysis and automating key diagnostic steps, AI can reduce diagnostic times and optimize patient outcomes. As AI-based tools continue to evolve and gain clinical validation, their integration into everyday practice will likely lead to a more efficient and accurate care for patients with valvular heart disease.

Learning points

- Artificial intelligence (AI) can improve the assessment of valvular heart disease by automating and averaging measurements such as peak velocities, pressure gradients, and other prognostic parameters (such as global longitudinal strain), which reflect the haemodynamic impact of the disease.
- Despite current limitations in echocardiography (e.g. poor acoustic window and analysis of uncommon pathological conditions), AI should be integrated into routine clinical practice to reduce diagnostic time without sacrificing accuracy.

## Introduction

Bicuspid aortic valve (BAV) is the most common congenital cardiac anomaly, affecting ∼1.3% of the general population.^1^ The bicuspid anatomy is more susceptible to degeneration, and the main complication of BAV is valve dysfunction, either in the form of aortic stenosis (AS) or aortic regurgitation (AR). Bicuspid aortic valve is also associated with proximal aortopathy and its rare but potentially fatal complications, such as aortic aneurysm and dissection.^2^ Although patients with BAV have a survival similar to that of the general population, it is estimated that ∼28% of them undergo aortic valve replacement (AVR) or repair during their lifetime.^3^

Echocardiography represents the first-line imaging modality not only for the diagnosis of BAV but also for assessing valvular anatomy and function. It plays a critical role in planning the surgical or transcatheter strategy and can predict the repairability of a BAV with significant regurgitation. In case of favourable anatomical conditions, repair may represent a very effective and durable surgical option. Two-dimensional (2D) and three-dimensional (3D) transoesophageal (TOE) echocardiography are essential to predict the feasibility of valve repair.^4^

Although still in its beginning, the use of artificial intelligence (AI) in echocardiography has seen exponential growth in recent years. It can be applied at different stages of image acquisition and interpretation, allowing for a faster diagnostic process and surgical planning without sacrificing accuracy. On larger scale, machine learning models can also predict valvular heart disease (VHD) progression rates, thus personalizing the patients’ management (follow-up interval, estimating timing to intervention).

Artificial intelligence-based software typically requires minimal or no user interaction. They are fully automated and promote standardization, often at the expense of flexibility. They should be distinguished from semi-automated software, which requires human intervention—for instance, manually adjusting contours. Several AI-based tools are now available for evaluating valve structure and function. They are capable of fully automated estimation of peak velocities and pressure gradients, fundamental in assessing the severity of valvulopathies, and 3D reconstruction of valves and surrounding structures, greatly improving pre-procedural planning. Artificial intelligence models, particularly those based on deep learning, showed good agreement with manual measurements for quantifying left ventricular volumes and function,^5,6^ which are crucial when evaluating how valve disease impacts the overall heart function. Artificial intelligence is also routinely used for estimating parameters that help to better define the patient’s prognosis and expected post-surgical outcome, such as global longitudinal strain (GLS).^7^

There are no contraindications to the use of AI in echocardiography for the diagnosis and management of patients with VHD. The main limitation is a poor acoustic window, which can significantly affect data interpretation. Suboptimal results may also occur in rarer pathological conditions (e.g. complex congenital cardiomyopathies), for which the AI algorithms may not have been adequately trained. In these cited cases, conventional manual measurements, calculations, and adjustments remain essential to ensure accuracy and clinical reliability.

In this case series, we present two clinical cases in which the use of different applications of AI in echocardiography helped to accurately diagnose the valvular dysfunction resulting from BAV and to tailor the appropriate intervention to the patients.

## Summary figure

**Figure:**
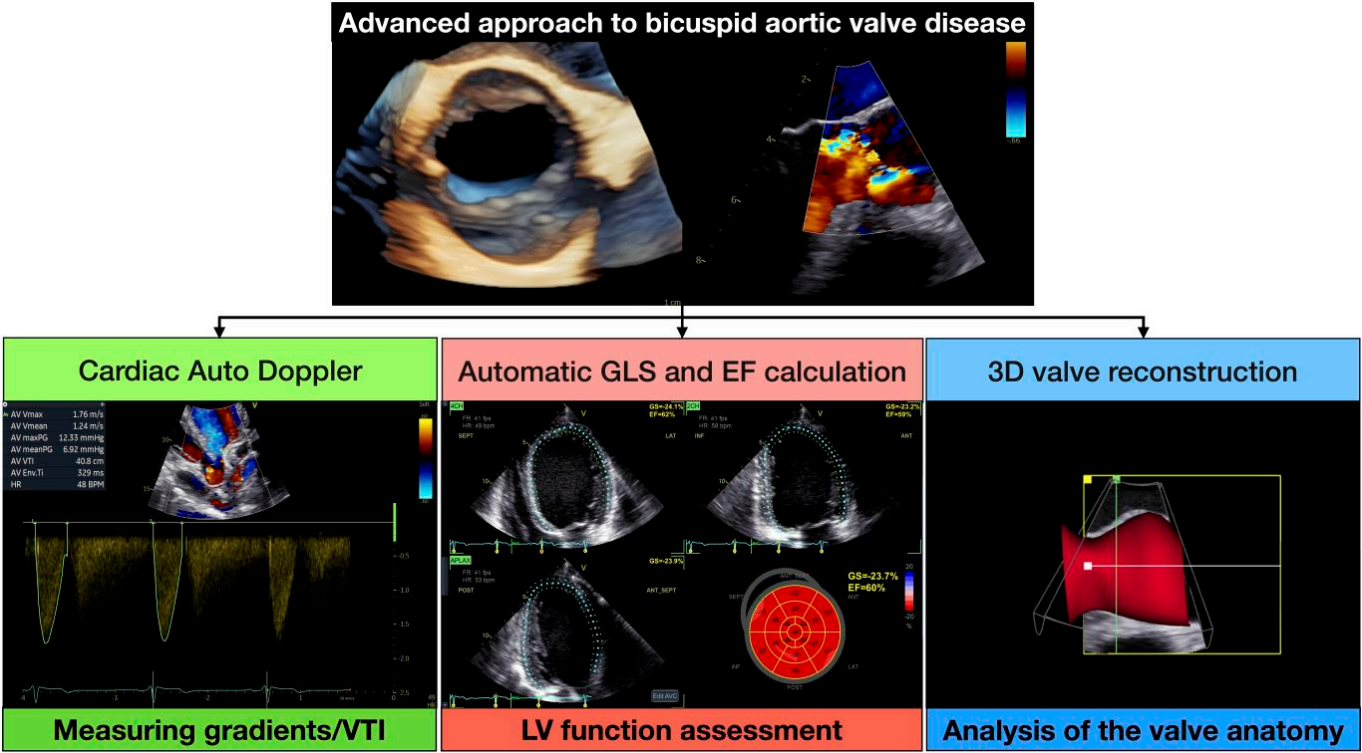
Comprehensive AI-powered approach to bicuspid aortic valve disease: Cardiac Auto Doppler to estimate gradients and velocity-time integrals (VTIs), automatic global longitudinal strain (GLS) and ejection fraction (EF) calculation to assess left ventricular (LV) function and three-dimensional (3D) valve reconstruction for a deep valve anatomical analysis.

## Case presentations

### Patient 1

**Figure 1:**
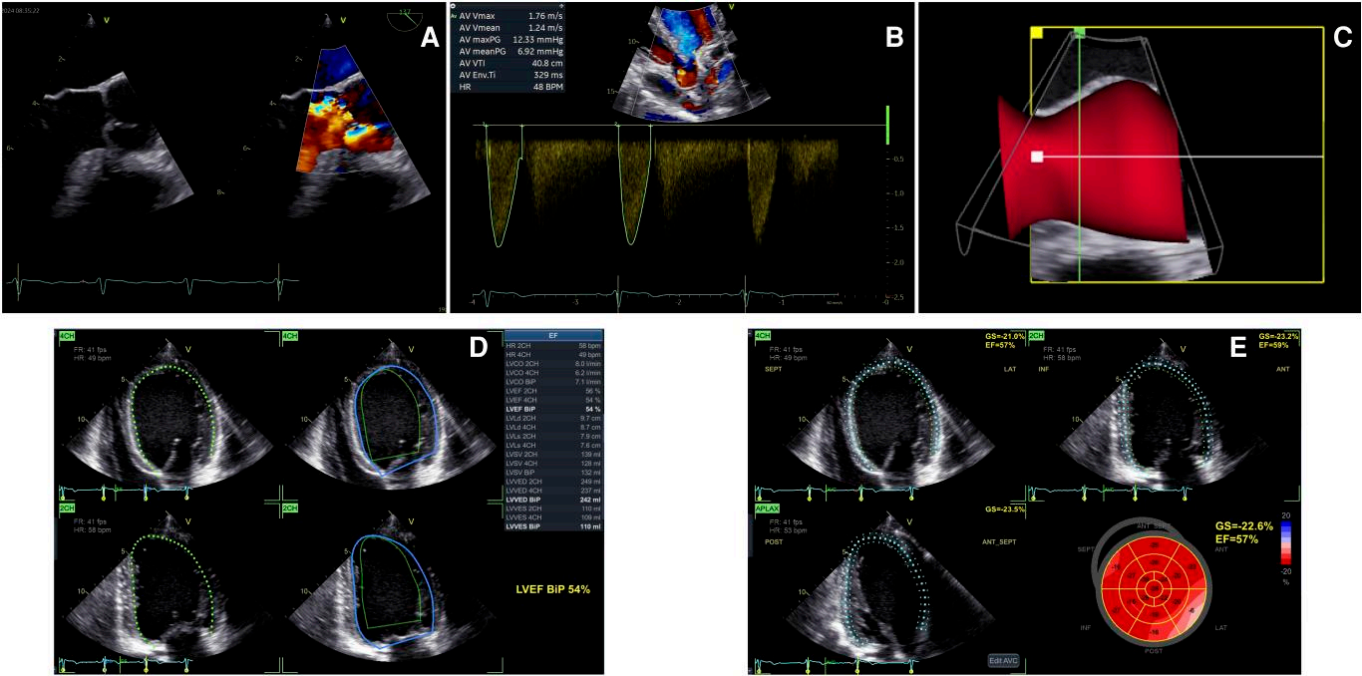
Artificial intelligence-powered echocardiographic assessment of a patient with bicuspid aortic valve and severe aortic regurgitation due to prolapse. (*A*) Prolapse of the anterior portion of the fused cusp causing severe eccentric aortic regurgitation. (*B*) Cardiac Auto Doppler function (GE HealthCare) to sample and average transaortic gradients to exclude aortic stenosis. (*C*) Semi-automated software (aortic valve quantification, GE HealthCare) three-dimensional reconstruction of the aortic root complex. (*D*) Automatic left ventricular volumes and ejection fraction estimation (Auto-EF, GE HealthCare) revealing a severe dilation and an ejection fraction at the lower normal limit. (*E*) Automatic global longitudinal strain calculation (Easy-AFI) demonstrating normal longitudinal contractility. AI, artificial intelligence; AR, aortic regurgitation; AVG, aortic valve quantification; 3D, three-dimensional; EF, ejection fraction; GLS, global longitudinal strain.

We present the case of a 17-year-old male football player, who was completely asymptomatic and reported no exercise limitations. He had no past medical history, no family history for cardiovascular disease, and was not taking any medical treatment. During a competitive sports medical examination, a diastolic heart murmur was detected. A transthoracic echocardiography (TTE) was recommended, revealing a significant AR in a BAV. Consequently, he was referred to our tertiary centre for further investigation. A TTE was initially repeated, which showed a ‘fused BAV’ phenotype with three sinuses of Valsalva and fusion of right and left coronary cusps with a median raphe. A severe eccentric AR, directed towards the anterior mitral leaflet, was observed. The left ventricle (LV) was found to be severely dilated, with an EF at the lower normal limit, according to AI-automated software measurement (Auto-EF software, GE HealthCare, Chicago, IL): LV end-diastolic volume index 138 mL/m*^2^*, LV end-systolic volume index 63 mL/m^2^, LV end-diastolic diameter index 34 mL/m^2^, LV end-systolic diameter index 22 mm/m*^2^*, and EF in Simpson biplane 54% (*Figure 1*). Another AI-based software (Easy-AFI), capable of autonomously recognizing the different echocardiographic windows and identifying the edges of the endocardium, calculated automatically GLS (GLS average −22.6%) (*Figure 1*). No contractility abnormalities were detected. The aortic root and tubular ascending aorta had normal dimensions. Subsequently, a TOE echocardiography was performed. A prolapse of the anterior portion of the fused cusp causing severe eccentric AR [effective orifice area (EROA) 0.48 cm^2^, regurgitant volume (RV) 84 mL] was noticed (*Figure 1*). The prolapse extended from the raphe to the anterior commissure, while the posterior portion of the cusp showed adequate coaptation (coaptation height 6 mm); initial fibrosis and retraction of the fused cusp (geometric height 13 mm) was also involved in the mechanism of AR. The commissural orientation was symmetric (angle between the non-fused commissures and the centre of the valve: 161°). Using a semi-automated software [aortic valve quantification (AVQ), GE HealthCare], a 3D reconstruction of the aortic root complex was quickly generated (*Figure 1*). The software provides an automatic alignment and segmentation of the left ventricular outflow tract (LVOT) and aortic annulus, automatically drawing and dimensioning the annular contour, with an option for manual adjustment by the operator.^8^ The aortic annulus resulted to be elliptical and slightly dilated (minimum diameter 22.9 mm, maximum diameter 28.6 mm, mean diameter 25.7 mm, annular circumference 81.1 mm, annular area 5.1 cm^2^). The LV dilation (LV end-systolic diameter index > 20 mm/m^2^) represented a surgical indication according to the latest European guidelines (Class IIb), and the valve was judged to have a good probability of repair.^9^ An aortic valve repair with annuloplasty (Biostable HAART Bicuspid 21, Corcym Inc., Englewood, CO), anterior cusp plication, and pericardial patch positioning (to increase the coaptation surface) was performed via minimally invasive approach (ministernotomy). The post-operative course was characterized by a rapid recovery. At the pre-discharge TTE, we observed normal aortic valve dynamics with no residual AR; a positive remodeling of the left ventricle (LV end-diastolic volume index 88 mL/m^2^) was already present. After about 3 months, he was asymptomatic and able to resume playing competitive football.

### Patient 2

**Figure 2:**
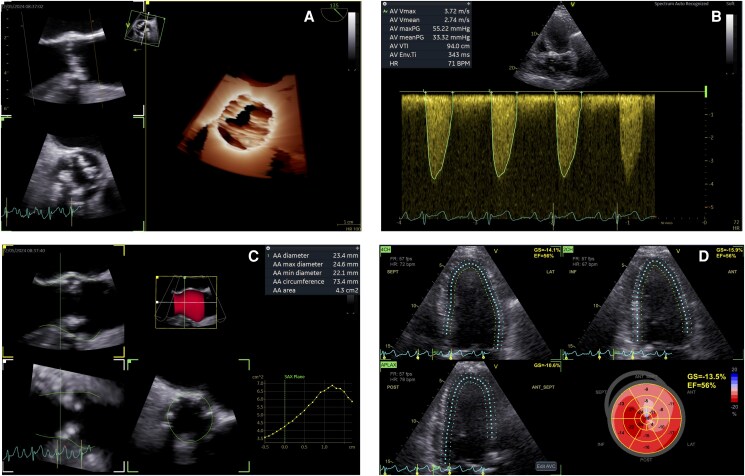
Artificial intelligence-powered echocardiographic assessment of a patient with a degenerated bicuspid aortic valve. (*A*) Three-dimensional echocardiography showing a stenotic ‘fused bicuspid aortic valve’ phenotype with fusion of the right and left coronary cusps. (*B*) Cardiac Auto Doppler function (GE HealthCare) to sample and average transaortic gradients (peak velocity 3.72 m/s, peak gradient 55 mmHg, mean gradient 33 mmHg). (*C*) Semi-automated aortic valve quantification software (GE HealthCare) used to determine the transcatheter bioprosthesis sizing. (*D*) Automatic global longitudinal strain calculation (Easy-AFI, GE HealthCare) demonstrating a significant systolic dysfunction (global longitudinal strain average −13.5%). AI, artificial intelligence; 3D, three-dimensional; BAV, bicuspid aortic valve; AVQ, aortic valve quantification; GLS, global longitudinal strain.

A 68-year-old male patient had been followed up for 4 years due to a degenerative aortic valvular disease. He had a history of hypertension and Stage IIIB chronic kidney disease. He was receiving only antihypertensive treatment. Due to a suspicion of severe AS, he was referred to our centre. During our evaluation, he reported to be asymptomatic, although he had a sedentary lifestyle. A TTE was performed raising suspicion of a calcified BAV. An AI-powered automated software (Cardiac Auto Doppler function, GE HealthCare) enabled the sampling and averaging of transaortic gradients and LVOT VTI over three beats, thereby calculating the aortic valve area (AVA) using the continuity equation: peak velocity 3.72 m/s, peak gradient 55 mmHg, mean gradient 33 mmHg, and AVA 1.25 cm^2^ (*Figure 2*). A moderate AR was associated. The LV was moderately dilated (LV end-diastolic volume index 87 mL/m^2^) and hypertrophic, and the left atrium was mildly dilated (left atrial volume index 40 mL/m^2^). The Easy-AFI software highlighted a preserved EF (56%), while GLS was significantly reduced (−13.5%) (*Figure 2*). To clarify the anatomy and severity of the valvular defect, a TOE echocardiography was performed, confirming a ‘fused BAV’ phenotype with three sinuses of Valsalva, fusion of the right and left coronary cusps with a short median raphe, a markedly hypomobile fused cusp, and reduced mobility of the remaining cusp (*Figure 2*). The 3D planimetric AVA was 1.0 cm². The associated AR was estimated as moderate-to-severe (vena contracta 6 mm, EROA 0.28 cm^2^, RV 38 mL). Given the haemodynamic impact of the valvular disease—evidenced by LV dilation, reduced GLS,^10^ and a dilated left atrium^11^—a surgical AVR was advised.

Due to the patient’s renal impairment, a total-body computed tomography (CT) without contrast was performed as part of the pre-operative planning for aortic valve surgery. An incidental intracranial mass, suspected to be malignant, was identified. During the multidisciplinary discussion, the transcatheter aortic valve implantation (TAVI) was chosen to expedite the neurosurgical treatment. Therefore, to determine prosthesis sizing without contrast media exposure, advanced TOE data were obtained using the AVQ semi-automated software: aortic annulus diameter, minimum 22.1 mm, mean 23.4 mm, and maximum 24.6 mm, and annular circumference 73.4 mm (*Figure 2*).^12,13^ A self-expanding Accurate Neo 2 size M valve (Boston Scientific, Marlborough, MA) was successfully implanted. At the 1-month follow-up, a TTE revealed a normal prosthetic function (mean gradient 6 mmHg, EROA 1.8 cm^2^). A trivial residual anterior paravalvular leak was detected. The patient was asymptomatic and in good functional status and was therefore able to undergo neurosurgical intervention to remove the intracranial mass.

## Discussion

Bicuspid aortic valve frequently leads to valve degeneration resulting in AS or AR requiring surgical treatment.

Diagnosis of this condition and subsequent management planning are mostly achieved by echocardiography. Of all cardiac imaging modalities, echocardiography is the most susceptible to inter-observer variability and is largely dependent on operator experience. It does not always show excellent reproducibility in quantifying LV volumes and function and has inherent limitations in grading VHD.^14,15^ As highlighted in these clinical scenarios, AI may help physicians increase the accuracy of image generation and data interpretation in many ways (*Summary figure*). Regarding BAV, it can play a key role in assessing valve anatomy and haemodynamics. Automatic contour tracing used for estimating peak velocities and VTIs has shown consistent results compared with manual tracing, potentially reducing human error and leading to faster assessments^16^; it can be particularly useful in patients with atrial fibrillation, where measurements should be averaged on at least five beats according to current guideline recommendations. Semi-automated 3D reconstruction of the LVOT and aortic valve showed promising results regarding the choice of prosthesis size and annular dimensions, especially in patients who cannot undergo contrast-enhanced CT scan, although further research is needed in this field.^14^

When evaluating a patient with BAV, detecting cardiac changes that occur in response to VHD may be informative on disease severity and represent an indication for intervention. In addition to already established parameters (LV end-systolic diameter > 50 mm/25 mm/m^2^ and EF ≤ 50% for asymptomatic AR and EF < 50% for asymptomatic AS), the focus in recent years has shifted towards more sensible analysis indicative of subclinical dysfunction, such as GLS. A pivotal meta-analysis^10^ evidenced how reduction in GLS < −14.7% is a fundamental parameter for prognostic classification of patients with asymptomatic AS and preserved EF. Several studies have demonstrated a high degree of accuracy in the estimation of cardiac chamber volumes^17^ and EF^18^ by fully automated software based on machine learning, and a recent work conducted using a convolutional neural network (CNN), showed how completely automated GLS measuring both increases the reproducibility of results and is less time consuming.^19^ This can be of paramount importance in expanding the use of this assessment, allowing for a better stratification of patients and detection of those who could benefit from an early intervention.

## Conclusions

The use of AI in echocardiography is only in its beginning, and yet it has already proven useful in several respects. Bearing in mind its limitations, the main ones being scarce worldwide availability and dependence on image quality, its use in everyday clinical practice should be encouraged, eventually leading to shorter diagnostic times and better patient care.^20^

## Lead author biography


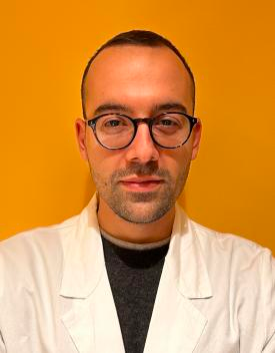


Dr Tommaso Viva graduated in medicine at the University of Pisa and completed the cardiology board examination at the University of Milan. He spent 1 year in the Echo Laboratory of the Cardiology Department of the Liege University Hospital, where he had the opportunity to focus and develop his skills in all the echo modalities. During this period, he was also involved in research programmes on valvular heart disease. He is fully certified in Adult Transthoracic and Transoesophageal Echocardiography (EACVI/EACTAIC Certification). He regularly holds courses in advanced echocardiography.

## Data Availability

The data underlying this article will be shared on reasonable request to the corresponding author.
